# Constraining Suprathermal Electron Evolution in a Parker Spiral Field With Cassini Observations

**DOI:** 10.1029/2020JA028669

**Published:** 2021-06-09

**Authors:** G. A. Graham, M. R. Bakrania, I. J. Rae, C. J. Owen, A. P. Walsh, M. J. Owens

**Affiliations:** ^1^ European Space Astronomy Centre (ESAC) European Space Agency (ESA) Villanueva de la Cañada Madrid Spain; ^2^ Department of Space and Climate Physics Mullard Space Science Laboratory University College London Dorking Surrey UK; ^3^ Department of Mathematics Physics and Electrical Engineering Northumbria University Newcastle upon Tyne UK; ^4^ Department of Meteorology University of Reading Reading UK

**Keywords:** Cassini, electrons, empirical modeling, interplanetary magnetic field, solar wind

## Abstract

Suprathermal electrons in the solar wind consist of the “halo,” present at all pitch angles, and the “strahl” which is a field‐aligned, beam‐like population. Examining the heliospheric evolution of strahl beams is key to understanding the in‐transit processing of solar wind suprathermal electrons, in particular, to identify electron scattering mechanisms and to establish the origin of the halo population. Not only does this have significant implications with regard to the kinetic processes occurring within the solar wind but also its thermodynamic evolution, as the suprathermal electrons carry the majority of the solar wind heat flux. In this investigation, an established model for suprathermal electron evolution in a Parker spiral interplanetary magnetic field is adapted from its original use. The model is constrained using solar wind strahl observed by the Cassini mission on its interplanetary journey to Saturn. The effects of large scale IMF geometry due to different solar wind velocities and application of different electron scattering factors are examined. It is found that slow solar wind speeds provide the closest match to the strahl width observations, both in terms of radial distance and electron energy trends, and that predominantly slower solar wind speeds were therefore likely observed by the Cassini mission en‐route to Saturn. It is necessary to include a strahl scattering factor which increases with electron energy in order to match observations, indicating that the strahl scattering mechanism must have an inherent energy dependence.

## Key Points

- Strahl broadening with distance is investigated using a model constrained by Cassini cruise phase observations
- Effects of solar wind speed/IMF length, scattering magnitude, and scattering energy relation are explored
- Cassini strahl observations are likely explained by an energy‐dependent scattering mechanism, the effect of which increases with energy

## 1 Introduction

Solar wind electrons consist of a thermal component population known as the core and suprathermal electrons, which generally comprise of a relatively isotropic population known as the halo, and a field‐aligned, beam‐like population known as strahl (e.g., Feldman et al., 1975). Suprathermal electrons are responsible for supporting the electric field required to maintain zero net charge in the solar wind (e.g., McComas et al., 1992) and for carrying the heat flux conducted into the solar wind from the corona (e.g., Pilipp, Miggenrieder, Montgomery, et al., 1987).

Strahl electrons typically travel away from the Sun along the interplanetary magnetic field (IMF) direction, although certain IMF typologies, such as local inversion in the field or closed loops associated with ICMEs, can result in observation of a sunward or bi‐directional strahl (e.g., Feldman et al., 1975; Gosling et al., 1994; Pilipp, Miggenrieder, Mühlhäuser, et al., 1987). In the absence of other effects, an electron with a given energy traveling outwards along the IMF should conserve magnetic moment. Thus, as IMF field strength decreases with distance from the Sun as it expands outwards with the solar wind plasma, strahl electrons are subject to adiabatic focusing. This should result in the formation of a strongly collimated beam (e.g., Owens et al., 2008). However, observations have demonstrated that strahl have significantly broader pitch angle widths than expected for only adiabatic effects to be acting on the electrons. For example, at ∼1 AU the strahl beam width should narrow to <1° but strahl width is frequently observed to be >20° (e.g., Anderson et al., 2012; Graham, Rae, Owen, & Walsh, 2018). Hence, strahl electron evolution must be subject to scattering processes. Coulomb interactions are generally considered to be too weak to fully explain the strahl broadening observed in the solar wind, in particular, at higher electron energies, and larger heliocentric distances (e.g., Horaites, Boldyrev, Wilson, et al., 2017; Ogilvie et al., 2000). This suggests that additional scattering processes must be involved, such as wave‐particle interactions, of which there a number of possible candidates with different generation mechanisms (e.g., Chen et al., 2013; Gary et al., 1994; Hellinger et al., 2014; Saito & Gary, 2007b).

A number of studies have examined the evolution of strahl beam width with heliocentric radial distance. Using Ulysses data, Hammond et al. (1996) observed that strahl width broadens with heliocentric radial distance between 1 and 2.5 AU. Graham, Rae, Owen, Walsh, et al. (2017) later confirmed this increase in strahl pitch angle width with distance, while also extending the strahl width observational range to ∼1–5.5 AU by making use of Cassini observations en‐route to Saturn. In addition, the fractional density of strahl electrons relative to total electrons has been observed to decrease with heliospheric radial distance while that of the halo electrons increases (e.g., Maksimovic et al., 2005; Stverak et al., 2009). This strahl‐halo density relation, in conjunction with strahl broadening with radial distance, suggests that strahl electrons are likely scattered to form some part of the halo population.

The in‐transit processing of strahl electrons is affected by both large‐scale IMF geometry (e.g., Fazakerley et al., 2016) and kinetic‐scale interactions (e.g., Gurgiolo et al., 2012). Thus, improved understanding of strahl evolution can not only provide further details into the thermodynamics of the solar wind but also provide valuable information regarding IMF topology and connectivity, and the small scale interactions which occur within the solar wind.

## 2 Motivation

Strahl width is observed to be highly variable at a given radial distance. For example, it has been shown that at 1 AU, strahl widths can lie anywhere between the limits of the instrument pitch angle resolution and isotropy (Anderson et al., 2012). However, on average, the increase in strahl beam width with heliocentric distance is relatively constant beyond 1 AU (Graham, Rae, Owen, Walsh, et al., 2017; Hammond et al., 1996). Using this average linear strahl width against distance relation, strahl broadening per unit radial distance can be found for each electron energy. Hammond et al. (1996) calculated the strahl broadening per AU for Ulysses observations out to ∼2.5 AU. Equation 1 describes the empirically derived relationship between strahl broadening per unit radial distance and electron energy. This equation shows a linear decrease in strahl broadening per unit radial distance with electron energy, suggesting that the strahl scattering process is energy dependent, with higher energy strahl being scattered less that lower energies.

(1) $$\frac{d\left(FWHM\right)}{dR}\;=\;30\left({}^{\circ}/AU\right)-0.1E\left({}^{\circ}/AU/eV\right)$$

where *R* is the heliospheric radial distance in units of AU, *E* is electron energy in units of eV and full‐width‐half‐maximum (FWHM) is a measure of strahl beam width. In Hammond et al. (1996), FWHM values were obtained by fitting a Gaussian function to each observed pitch angle distribution at a given electron energy, for a given radial distance. The Gaussian function also included a background term, to account for the suprathermal halo component of the electron distribution, and it was required that the peak signal be at least two times greater than the background to be included as strahl in their analysis.

Owens et al. (2008) developed a model to examine the evolution of suprathermal electron pitch angle distributions along open Parker spiral IMF lines that used the solar wind strahl observations reported in Hammond et al. (1996) as constraints. In this model, two processes were applied to the strahl pitch angle distribution as it evolved: adiabatic focusing and an “ad‐hoc” pitch angle scattering factor, which was assumed to be constant with heliospheric radial distance, electron kinetic energy, and time (see Section 3 for further details). This model demonstrated the pertinent effect that the IMF geometry can have on suprathermal electron evolution, in particular producing two distinct regions. The first, an inner region where the IMF is mostly radial, in which the effect of adiabtic focusing dominates and results in the formation of a narrow strahl beam by ∼0.1 AU. The second, an outer region where the IMF becomes more spiraled, in which the effect of pitch angle scattering dominates and results in the strahl beam broadening significantly beyond ∼0.5 AU. In this study, we are concerned with the region in which scattering dominates, as the observations we are investigating are from ∼1 AU and beyond. However, it should be noted that for regions closer to the Sun, < 0.7 AU, a slight decrease in the strahl width with the radial distance has been observed (Berčič et al., 2019). More specifically, this relation was found for lower energy strahl in solar wind with low values for the parallel component of the core electron beta (*β*_*ec*_ = 2*μ*_0_ *n*_*ec*_ *k*_*B*_ *T*_*ec*∥_/*B* ^2^), i.e., in solar wind that is more stable to kinetic instabilities and should therefore experience less scattering (this is discussed further in Section 5).

The modeled effect of scattering produced an approximately linear increase in strahl width beyond ∼0.5 AU. Thus the Owens et al. (2008) model was able to closely match the Ulysses observations of average strahl width at a given heliospheric radial distance. The energy relationship found by Owens et al. (2008), by matching to the radial trend observed by Hammond et al. (1996) using a constant scattering factor, is given in Equation 2. This modeled energy dependence of strahl broadening is much weaker than for the empirically derived dependence shown in Equation 1.

(2) $$\frac{d\left(FWHM\right)}{dR}\;=\;17\left({}^{\circ}/AU\right)-0.013E\left({}^{\circ}/AU/eV\right)$$

The energy dependence of strahl broadening given in Equation 2 arises solely from the time‐of‐flight effects of the electrons. In the presence of a constant rate scattering mechanism with no relation to electron energy, strahl broadening per unit radial distance should decrease with electron energy (Owens et al., 2008). Since higher energy electrons travel a greater radial distance per unit of time and should therefore experience greater adiabatic focusing. Thus, although the observed radial trend could be matched, the modeled relationship between strahl broadening per unit radial distance and electron energy does not correspond to the Hammond et al. (1996) observations; this is consistent with the possibility of a strahl scattering process which is energy dependent.

A more recent observational investigation by Graham, Rae, Owen, Walsh, et al. (2017) found strahl widths and calculated the strahl broadening per AU in the same manner as Hammond et al. (1996). However, the observations were made by the Cassini spacecraft and extended out to ∼5.5 AU. Equation 3 describes the empirically derived relationship between strahl broadening per unit radial distance and electron energy.

(3) $$\frac{d\left(FWHM\right)}{dR}\;=\;17.7\left({}^{\circ}/AU\right)+0.0034E\left({}^{\circ}/AU/eV\right)$$

This relationship is very different from that obtained by Hammond et al. (1996) and instead shows a slight increase in strahl broadening per unit radial distance with electron energy. This relationship suggests that the dominant scattering mechanism affects higher energy strahl more than lower energies. It should be noted that, although the increase with energy shown in Equation 3 is small, it has significant implications regarding the dominant scattering mechanism experienced by the strahl. Since, even for a constant modeled scattering rate, the opposite energy relation is expected.

The relationships observed by Hammond et al. (1996) and Graham, Rae, Owen, Walsh, et al. (2017) are both significantly different from each other and from the modeled relationship found by Owens et al. (2008). It is therefore important to consider the differences between the two sets of observations and the model. Hammond et al. (1996) used Ulysses data over a heliolatitude range of +30° to −50° whereas Cassini had a near‐equatorial trajectory and so the data used by Graham, Rae, Owen, Walsh, et al. (2017) had minimal latitude variations. Hammond et al. (1996) also examined intervals in the fast solar wind (∼660–860 km s^−1^), whereas Graham, Rae, Owen, Walsh, et al. (2017) did not obtain solar wind velocity information due to the instrumental limitations of the Cassini Plasma Spectrometer (Lewis et al., 2008; Young et al., 1998). Finally, Owens et al. (2008) used the Hammond et al. (1996) observations as constraints but, for the sake of simplicity, chose to model only 800 km s^−1^ solar wind for a constant heliolatitude.

In theory, the Parker spiral magnetic field becomes more loosely wound (or more radially oriented) as heliolatitude increases, which is in general agreement with IMF observations (Forsyth et al., 2002). The Parker spiral IMF is also more loosely wound (more radially oriented) for higher solar wind velocities. Hence, heliolatitude and solar wind speed may have an effect on the path length traveled by the field‐aligned strahl electrons. It is also important to consider the possible effects of the different solar origins and in situ properties of the solar wind plasma encountered by the Cassini and Ulysses spacecraft (e.g., Abbo et al., 2016; Xu & Borovsky, 2015, and references therein). Since different solar wind origins, e.g., coronal hole versus streamer‐belt regions, may result in different initial electron distributions or electrons that undergo differing degrees of scattering in‐transit within solar wind plasma with different characteristics. In order to investigate these possibilities, we implement and extend the Owens et al. (2008) model and use the Cassini observations reported in Graham, Rae, Owen, Walsh, et al. (2017) as constraints. We examine the modeled strahl widths for different distances and electron energies, while considering the effect of solar wind velocity, that is, average IMF geometry, as well as the effect of different scattering factors. Finally, the effect of including a scattering factor with an inherent energy dependence will be examined.

## 3 Method

We implement the Owens et al. (2008) model for a number of different solar wind velocities and degrees of strahl scattering, see Table 1. Below we provide a description of the model and how we make use of it within this study (for a more detailed discussion of the strahl evolution simulation we refer the readers to the original study).

**Table 1:** Parameters Used for the Simulation Runs in This Investigation

	*V*_*SW*_ (km s^−1^)	*σ*	*E* (eV)	*σ* Energy relation
A	300–1,000	0.0022	77	Constant
B	800	0.0022–0.0035	77–600	Constant
	450	0.002, 0.0022	”	”
	300	0.0015–0.0022	”	”
C	450	0.0019 at 77 eV	77–600	*σ* ∝ 10^−6^ eV^−1^ × *E*
	”	0.0022 at 77 eV	”	”
	300	0.0015 at 77 eV	”	”
	”	0.0017 at 77 eV	”	”

*Note*. VSW is the selected solar wind speed, σ is the applied scattering factor and *E* in the electron energy. Case A shows the values used for investigation of different solar wind speeds. Case B shows the values used for investigating different scattering factors for three different solar wind speeds. Case C shows the values used for investigation of a nonconstant scattering factor.

The radial velocity of a strahl electrons consists of the radial component of the electron propagation along the magnetic field (*V*_∥_) and the advection with the radially flowing solar wind (*V*_*SW*_). This can be written as:

(4) $$\begin{array}{ccc} V_{R} & = & V_{SW}\;+\;V_{∥}cos\left[\gamma \right]\\ & = & V_{SW}\;+\;\left[\sqrt{\frac{2E}{m_{e}}}cos\left[\alpha \right]\right]cos\left[arctan\left[\frac{2\pi }{T_{ROT}V_{SW}}Rcos\left[\theta \right]\right]\right] \end{array}$$

where *γ* is the angle between the magnetic field and radial direction (i.e., Parker spiral angle). *E*, *α*, *R*, *T*_*ROT*_ and *θ* represent the electron energy, electron pitch angle about the magnetic field direction, heliocentric distance, the Sun's rotational period, and the heliographic latitude, respectively.

In the absence of scattering effects, the evolution of *α* with *R* is controlled by conservation of magnetic moment:

(5) $$sin^{2}\left[\alpha \left(R\right)\right]\;=\;\frac{B_{TOT}\left(R\right)sin^{2}\left[\alpha \left(R_{0}\right)\right]}{B_{TOT}\left(R_{0}\right)}$$

where $B_{TOT}\left(R\right)$ is the magnetic field strength at distance *R* and *R*_0_ is a reference distance. Magnetic flux conservation implies that the radial component of the IMF strength falls off as 1/*R* ^2^ and, in the Parker spiral model of the solar wind, the azimuthal component of the magnetic field is given by $B_{\gamma }=B_{R}tan\left[\gamma \left(R,\theta \right)\right]$. The heliocentric distance and pitch angle of an electron at a given time *t* can thus be found by numerically integrating Equations 4 and 5. The strahl evolution simulation uses a uniform numerical grid in cosine pitch angle (*μ* = cos*α*) and heliocentric distance space. At the start of the simulation all grid cells are set to zero except at 1 *R*_*S*_ where an isotropic population of electrons with number density *N*_*INIT*_ is placed. For each time step, the new *R* and *μ* of each electron is calculated using Equations 4 and 5. When these new values fall between an *R* or *μ* then the electrons are split between the bounding grid cells by linear interpolation. Any electrons that propagate to the end of the simulation grid are lost.

The effect of pitch angle scattering is simulated using an “ad‐hoc” process in which the electrons within in each grid cell at each time step are pitch angle broadened by a Gaussian function of *μ*. Assuming that at time step *i* there are *N*_0_ electrons in the *μ* grid cell centered at *μ*_0_ then at time step *i* + 1 the electrons are spread in *μ* by the following equation:

(6) $$\frac{dN\left(\mu \right)}{d\mu }\;=\;\frac{N_{0}}{\sigma \sqrt{2\pi }}exp\left[-\frac{{\left(\mu -\mu_{0}\right)}^{2}}{2\sigma^{2}}\right]$$

where the number of electrons is conserved is given by,

(7) $$N_{0}\;=\;\int_{-1}^{1}d\mu \frac{dN}{d\mu }$$

If *σ* increases then the level of simulated scattering will also increase, as the electrons are spread over a larger range of *μ*. Hence, *σ* is referred to as the scattering factor. In this study, we are varying *σ* along with *V*_*SW*_ in order to match to the Graham, Rae, Owen, Walsh, et al. (2017) observations of strahl pitch angle width from ∼1 to 5.5 AU.

**Figure 1:**
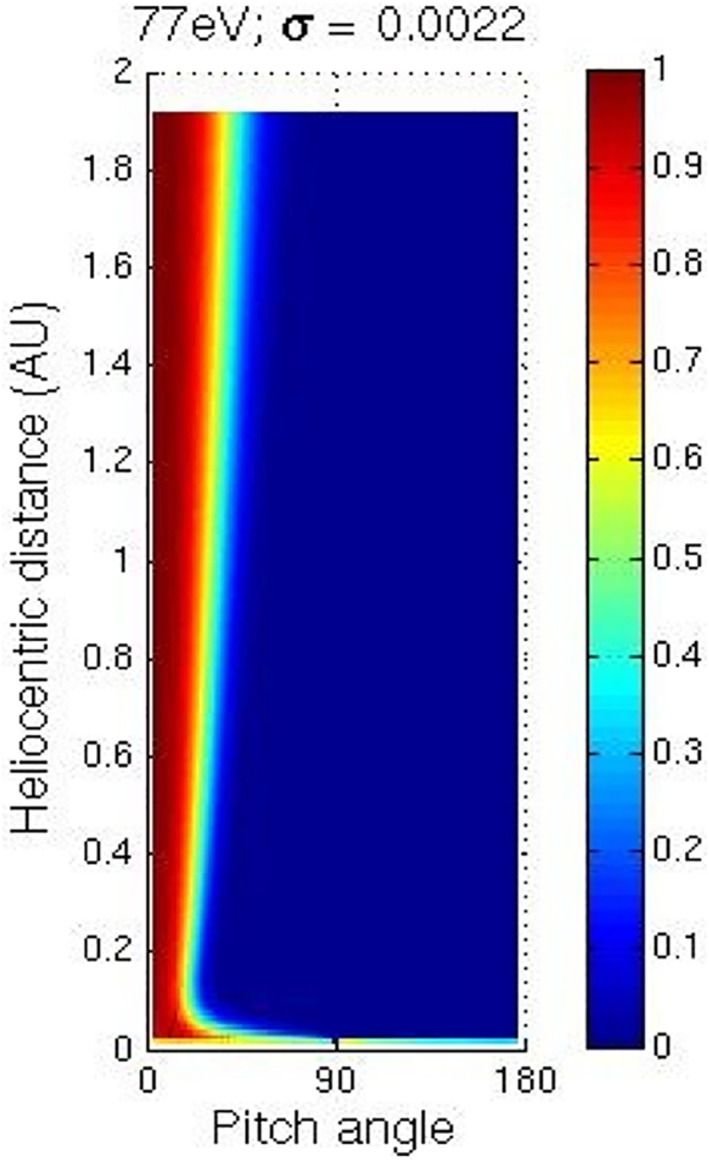
Results of a numerical simulation of suprathermal electron evolution with a pitch angle scattering factor of 0.0022 with for an initially isotropic distribution. The modeled solar wind speed and electron energy are 800 km^−1^ and 77 eV, respectively. Electron pitch angle is plotted against heliocentric radial distance. The color scale represents normalized suprathermal electron number density.

Following Owens et al. (2008), our initial chosen parameters include: a time step length of 100 s (dt), 0.01 AU radial grid spacing (dR), 500 pitch angle bins, a magnetic field strength of 5 nT at 1 AU and a heliolatitude of 0°. Each of these parameter choices was investigated at the beginning of this study and found to be suitable by inspection. Figure 1 shows an example run of the Owens et al. (2008) model, for an electron population that is initially isotropic. This example is for a modeled solar wind speed and electron energy of 800 km^−1^ and 77 eV respectively. The color bar represents the suprathermal electron number density, which has been normalized with respect to the maximum density at each heliocentric distance. The distribution of electrons broadens as heliocentric distance increases and the maximum density is always along a pitch angle of 0°. For each model run, the pitch angle width of the strahl is found for each radial distance bin by calculating the FWHM of the electron pitch angle distribution. This is achieved by fitting a function consisting of a Gaussian peak and constant background to the pitch angle distribution in the same manner as Graham, Rae, Owen, and Walsh (2018) Graham, Rae, Owen, Walsh, et al. (2017) and Hammond et al. (1996).

## 4 Results

### 4.1 Considering Higher Electron Energies

Table 1 summarizes the electron energies (77–600 eV), solar wind velocities (300–1000 km s^−1^), scattering factors (0.0015–0.0031) and scattering factor energy relations (constant and increasing with energy) for the different simulations runs presented in this study. Previous work using this model investigated energies of 77–225 eV in order to match the energy range of the Ulysses strahl observations (Owens et al., 2008). We have elected to use electron energies up to 600 eV, in order to match the energy range of the Cassini strahl observations.

**Figure 2:**
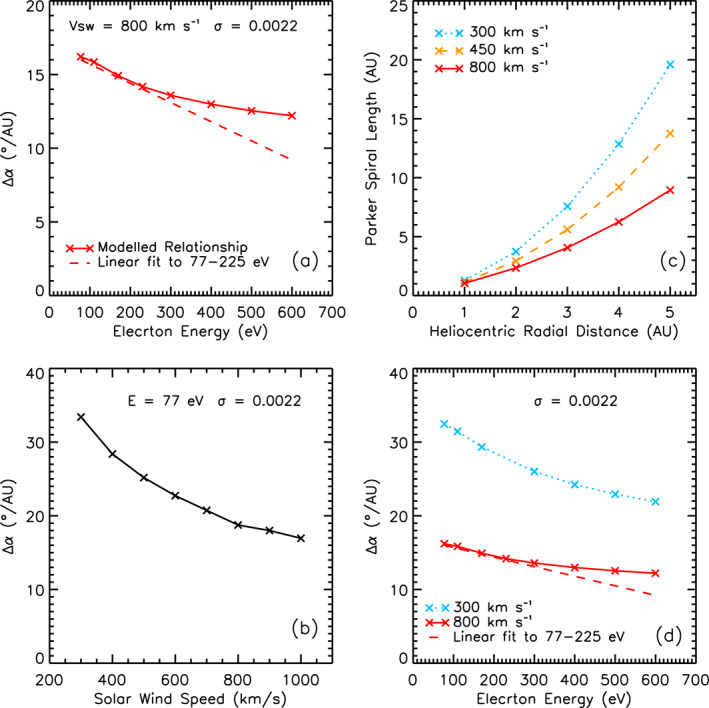
(a) Simulation results for variation of strahl width per unit distance as a function of electron energy. The results (solid line) show the energy relation obtained for simulations run for 800 km s^−1^ solar wind with a scattering factor of 0.0022. The relation shown by the dashed line is the extrapolation of the results reported in Owens et al. (2008) for 77–225 eV electrons. (b) Simulation results for variation of strahl width per unit distance as a function of solar wind velocity for an electron energy of 77 eV, a scattering factor of 0.0022. (c) Parker spiral length against heliocentric radial distance for 300 km s^−1^ (blue dotted line), 450 km s^−1^ (orange dashed line), and 800 km s^−1^ (red solid line). (d) Simulation results for variation of strahl width per unit distance as a function of electron energy for a scattering factor of 0.0022. The results are shown in blue (dotted line) are for a solar wind velocity of 300 km s^−1^. The results shown in red (solid and dashed lines) are the same as shown in (a).

Panel (a) of Figure 2 shows the modeled results for change in strahl width per unit radial distance against electron energy. Following Owens et al. (2008), these results were obtained for a solar wind speed of 800 km s^−1^ and an electron scattering factor of 0.0022; values that were originally selected as they produced results closest to the Hammond et al. (1996) observations of 77 eV strahl radial evolution (and also agree well with energies up to 225 eV). When we model the evolution of higher energy electrons, it can be seen that the pitch angle change per AU does not continue to decrease linearly with energy. This can be seen in Panel (a), in which, beyond ∼250 eV, the simulated energy relation for all electron energies (solid line) flattens out and departs from the linear relation given in Equation 2 (dashed line).

### 4.2 Solar Wind Velocity Observed by Cassini

In this study, the Owens et al. (2008) model is used to match to Cassini strahl observations from its interplanetary journey to Saturn (Graham, Rae, Owen, Walsh, et al., 2017). However, due to the field‐of‐view restrictions of the Cassini electron instrument, obtaining solar wind information is challenging and requires making significant assumptions (Lewis et al., 2008). Hence, Graham, Rae, Owen, Walsh, et al. (2017) were not able to obtain solar wind information for the Cassini strahl study. However, it should be noted that Cassini's interplanetary trajectory remained at low heliographic latitudes and was therefore likely mixed‐speed, but predominantly slow solar wind.

In August 1999, the Cassini spacecraft performed an Earth Flyby, during which time the ACE spacecraft was at L1 making observations of the solar wind upstream of Cassini. Examination of the magnetic field data of the two spacecraft revealed observations of similar magnetic features, observed by Cassini at Earth for the expected times based on solar wind speed observed by ACE in conjunction with the magnetic field information (Graham, 2018). In particular, a magnetic cloud was identified (smooth rotation of the magnetic field) which passed both spacecraft. Hence, feature matching was used to estimate the solar wind speeds seen by Cassini during Earth Flyby using upstream ACE solar wind velocity information. It was found that at ∼1 AU Cassini was subject to wind speed with a median of ∼530 km s^−1^, a minimum of ∼380 km s^−1^ and a maximum of ∼770 km s^−1^.

### 4.3 The Effect of Solar Wind Velocity

The strahl evolution simulation was run for a number of different solar wind velocities in order to further investigate the effect of IMF geometry on strahl evolution. Panel (b) of Figure 2 shows the modeled strahl width broadening per AU for solar wind speeds ranging from 300 to 1,000 km s^−1^. For each of the simulation runs an electron energy of 77 eV and a scattering factor of 0.0022 was implemented (see Case A in Table 1). We find that strahl width broadening per AU decreases with respect to solar wind velocity. This relationship is as expected since faster solar wind will have a more radial IMF. Panel (c) of Figure 2 demonstrates how Parker spiral IMF length increases with radial distance for different solar wind speeds. The increase in Parker spiral length with radial distance is smaller for faster wind speeds. Hence, electrons traveling along the IMF in fast solar wind will experience a greater change in radial distance and thus, a greater change in magnetic field strength per unit time than in the slow wind. In the case of a scattering rate that is constant with time and distance (as is modeled), this means that for a given time, the electron will experience greater focusing in the fast solar wind than the slow for the same scattering effect.

The effect of solar wind speed on IMF length also influences the observed energy relation for change in strahl width per AU. Panel (d) of Figure 2 shows the energy relation for slow (300 km s^−1^) and fast (800 km s^−1^) solar wind speeds. It can be seen that a beam of lower energy (slower) electrons experiences greater broadening per AU than higher energy (faster) electrons due to time‐of‐flight effects that is, a faster electron will experience a greater change in radial distance and magnetic field strength per unit time and therefore, experience greater adiabatic focusing effects. This energy relation is much steeper (approximately twice as steep) in the slow wind than the fast.

### 4.4 Applying a Greater Scattering Factor and Comparison to Cassini Observations

**Figure 3:**
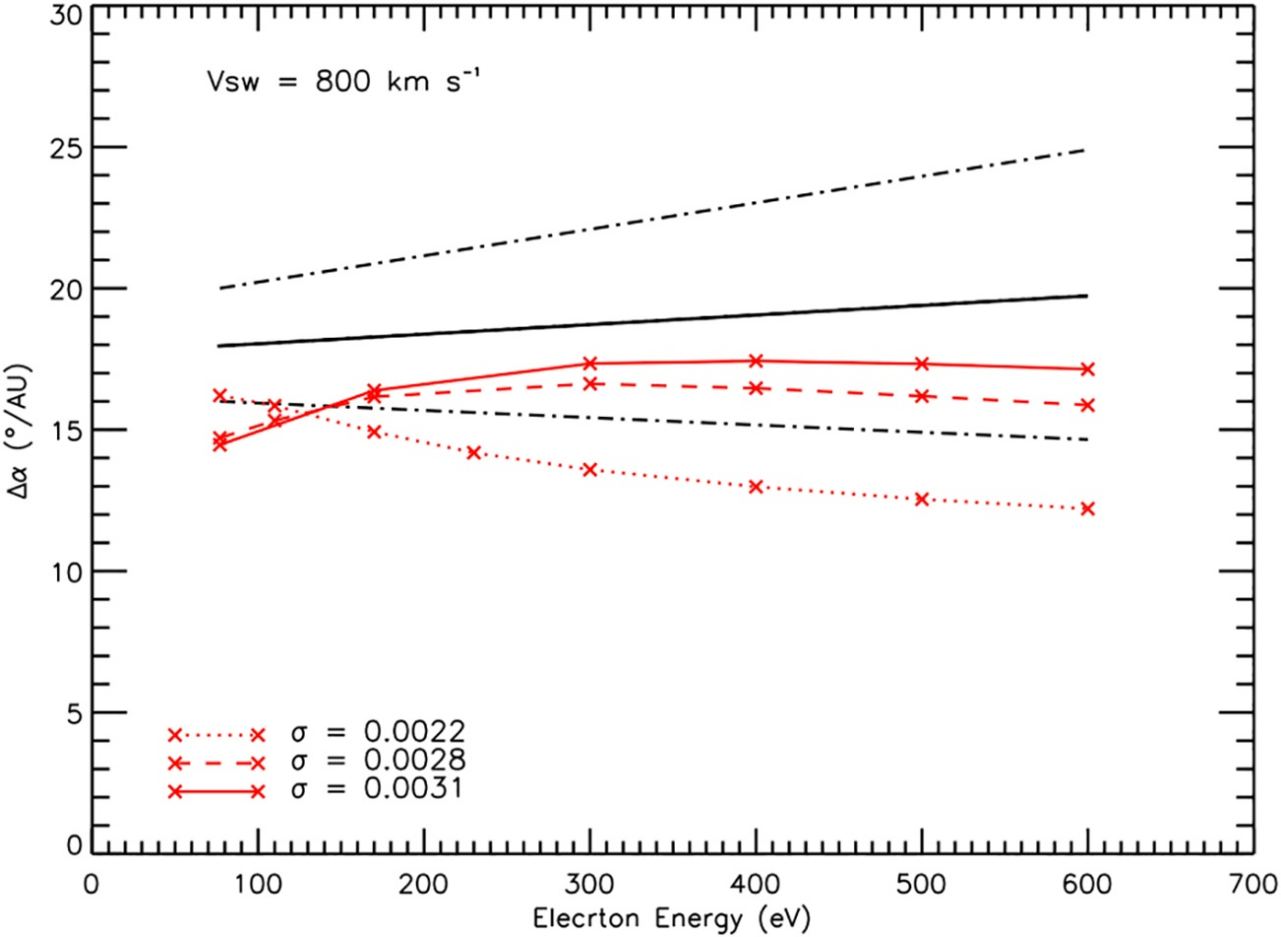
Simulation results for variation of strahl width per unit distance as a function of electron energy for a solar wind velocity of 800 km s^−1^. The results shown by the red solid line, dashed line, and dotted line are for a scattering factor of 0.0031, 0.0028, and 0.0022, respectively. The black solid line shows the fitted results from the Graham, Rae, Owen, Walsh, et al. (2017) observational study and the dot‐dash lines show the 1*σ* uncertainty for the fit.

Cassini observations of strahl beam width extended the heliocentric distance range from 1–2.5 to 1–5.5 AU and demonstrated that strahl width continues to increase with distance. However, Graham, Rae, Owen, Walsh, et al. (2017) found that strahl broadening per AU increased with electron energy as opposed to the decrease with energy modeled by Owens et al. (2008) and observed by Hammond et al. (1996). Figure 3 shows the effect of increasing the selected scattering factor for the simulation from 0.0022 to 0.0031, for a solar wind speed of 800 km s^−1^ and electron energies of 77–600 eV. We also extend the linear fitting range for strahl width with radial distance to 1–5.5 AU.

It can be seen that increasing the scattering factor to 0.0031 brings the simulated results for most electron energies within the uncertainty for the fits to the Graham, Rae, Owen, Walsh, et al. (2017) observations of strahl broadening per AU, shown by the dotted–dashed lines in Figure 3. In addition, when this alteration is applied to the simulations, the trend for broadening per AU with electron energy is also altered. Above 300 eV the decrease in strahl broadening per AU is less pronounced than the decrease as shown in Panel (d) of Figure 2 for *σ* = 0.0022; in fact, broadening per AU is almost uniform across the higher electron energies for increased scattering factor. Below 300 eV there is an increase in strahl broadening per AU with electron energy.

Increasing the scattering factor brings the simulated results within error of the fits to the energy relation observed by Cassini (Equation 3). However, a constant, larger scattering rate does not produce a strahl evolution which agrees with the radial distance relation. This is because increasing the scattering rate at lower electron energies, by the same amount as for higher energies, results in a strahl width at a given radial distance that is larger than the Cassini observations for low energy electrons. For example, using 800 km s^−1^ wind speed, a scattering factor of 0.0031 produces a strahl width for ∼77 eV electrons that is ∼40° greater than observed by Cassini at 1 AU (Graham, Rae, Owen, Walsh, et al., 2017).

**Figure 4:**
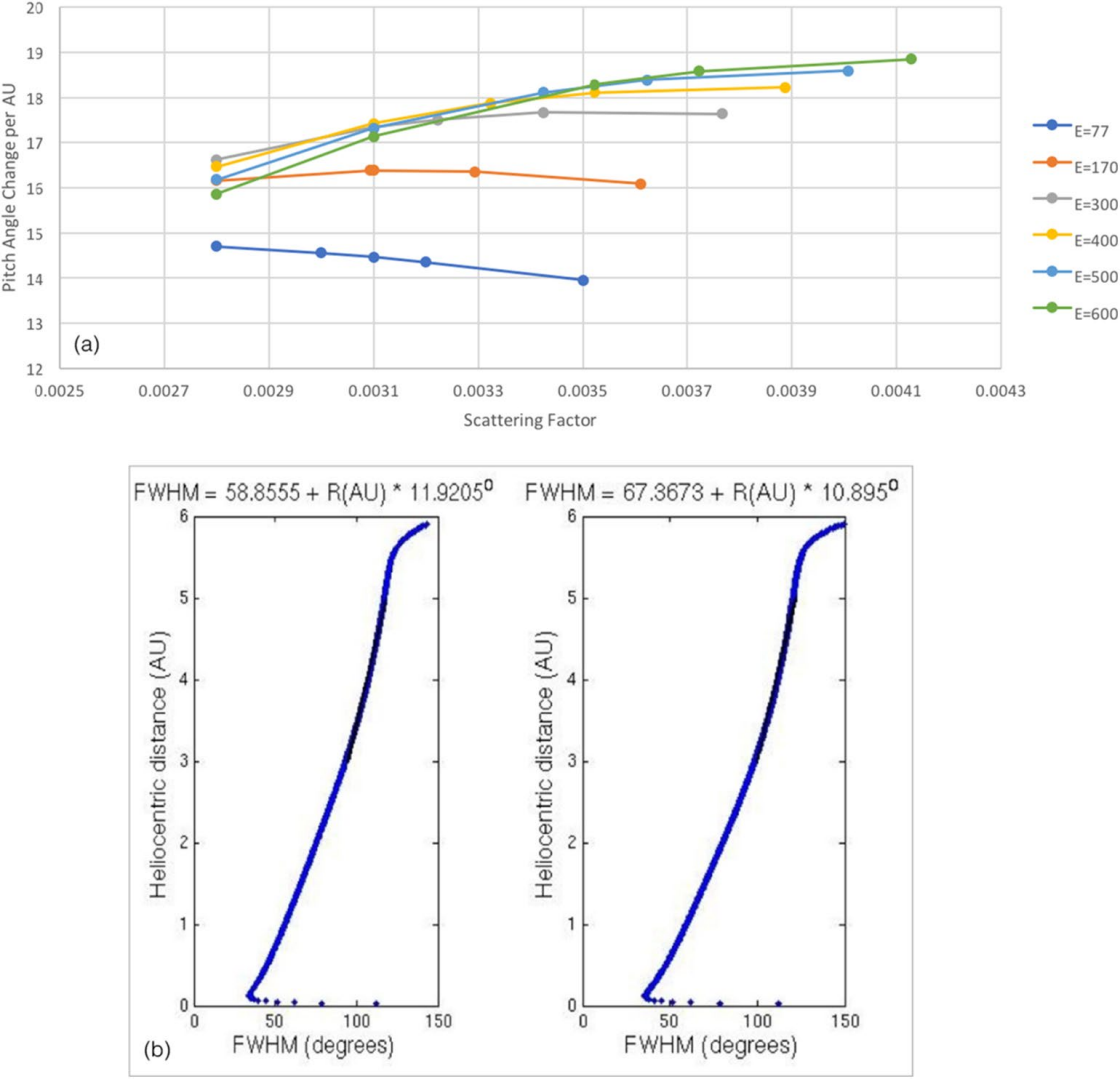
(a) Simulation results for variation of strahl width per unit distance as a function of scattering factor for electron energies ranging from 77 to 600 eV and a fitting range of 1–5.5 AU. (b) Results of a numerical simulation of suprathermal electron evolution with a pitch angle scattering factor of 0.0028 (left) and 0.0031 (right). FWHM of the electron pitch angle distribution is plotted against heliocentric radial distance. The equation above each plot is for a linear fit to the simulated results from 3 to 5 AU. The steep increase in pitch angle width near 6 AU is a result of the edge effects of the simulation.

Strahl broadening per AU against scattering factor for different electron energies is shown in Panel (a) of Figure 4. It was found that, for most electron energies, strahl broadening per AU correlated with applied scattering factor. However, the opposite trend was found for lower energy strahl (77 and 170 eV), with higher scattering factors resulting in a smaller value for strahl broadening per AU. In other words, applying a greater degree of scattering to the lower energy electrons results in a more gradual increase in strahl width with distance from 1 to 5.5 AU.

Panel (b) of Figure 4 shows the FWHM of the strahl beam against distance for 800 km s^−1^ solar wind and 77 eV electrons, with a scattering factor of 0.0022 (left) and 0.0031 (right). It can be seen that for higher scattering rates the strahl beam is broader within the region in which the effect of adiabatic focusing dominates (∼0–0.1 AU) and thus the simulated strahl is broader before the effects of scattering begin to dominate their evolution. The 77 eV strahl is also consistently broader across the radial range when using a higher scattering rate. However, the modeled results only produce an approximately linear relation of strahl width with distance and this becomes significant when large scattering rates are applied to lower energy electrons. As can be seen in Panel (b) of Figure 4, applying a scattering factor of 0.0031 results in a rate of change of strahl width that falls off at larger radial distances. Thus, linear fitting to the modeled trends with radial distance may not appropriate for low energy strahl when applying larger scattering factors.

### 4.5 Applying a Nonconstant Scattering Factor

The difference between modeled and observed energy relations for strahl beam width broadening per AU suggests that the scattering rate may not be constant with electron energy. Both the Ulysses and Cassini observations display a strahl broadening per AU energy relation that differs from the energy relation produced by a modeled constant scattering factor. In Graham, Rae, Owen, Walsh, et al. (2017) it was suggested that there may be a dominant strahl scattering mechanism with an inherent energy relation which could account for the observed difference between modeled and observed energy relations. From examination of the Graham, Rae, Owen, Walsh, et al. (2017) fits, it can be seen that a scattering factor that increases by 0.0001 per 100 eV would likely match observations. Thus, a scattering factor which increased with a gradient of 10^−6^ eV^−1^ for energies ranging from 77 to 600 eV was selected.

**Figure 5:**
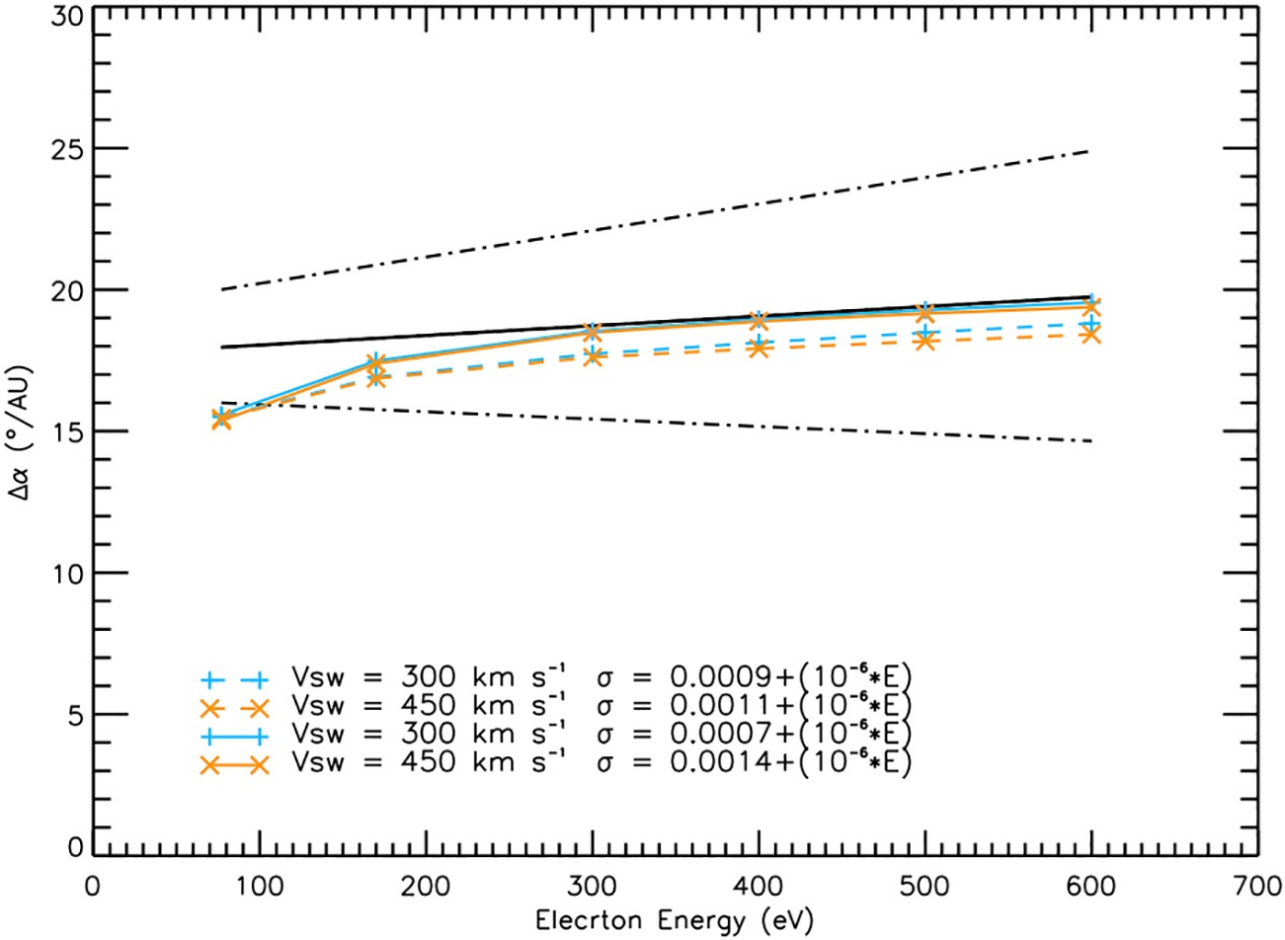
Simulation results for variation of strahl width per unit distance as a function of electron energy for a scattering factor which increases with electron energy. The black solid line shows the fitted results from the Graham, Rae, Owen, Walsh, et al. (2017) observational study and the dot‐dash lines show the 1*σ* uncertainty for the fit. The results shown in blue plus symbols (+) and orange crosses (x) are for a solar wind velocity of 300  and 450 km s^−1^ respectfully. For both solar wind speeds, the results shown by a solid line are for higher applied scattering factors than for the results shown by a dashed line.

Figure 5 shows the results for a 300  and 450 km s^−1^ solar wind speed. Greater scattering factors were applied to the 450 km s^−1^ wind speed runs than the 300 km s^1^ runs (see Case C of Table 1), since strahl in faster solar winds experiences a greater adiabatic focusing effect and so a greater scattering factor is required to match the Graham, Rae, Owen, Walsh, et al. (2017) observations. We have also excluded 800 km s^−1^ wind speeds as the higher scattering factors required do not agree with the radial trends observed (see Section 4.4). The results for energies above ∼ 150 eV for all three wind speeds lie within the upper and lower bounds of the (Graham, Rae, Owen, Walsh, et al., 2017) 1 sigma uncertainties. It can also be seen that for electrons with energies greater than ∼300 eV, the simulation results match very closely to the Graham, Rae, Owen, Walsh, et al. (2017) best fit to the data.

## 5 Discussion

**Figure 6:**
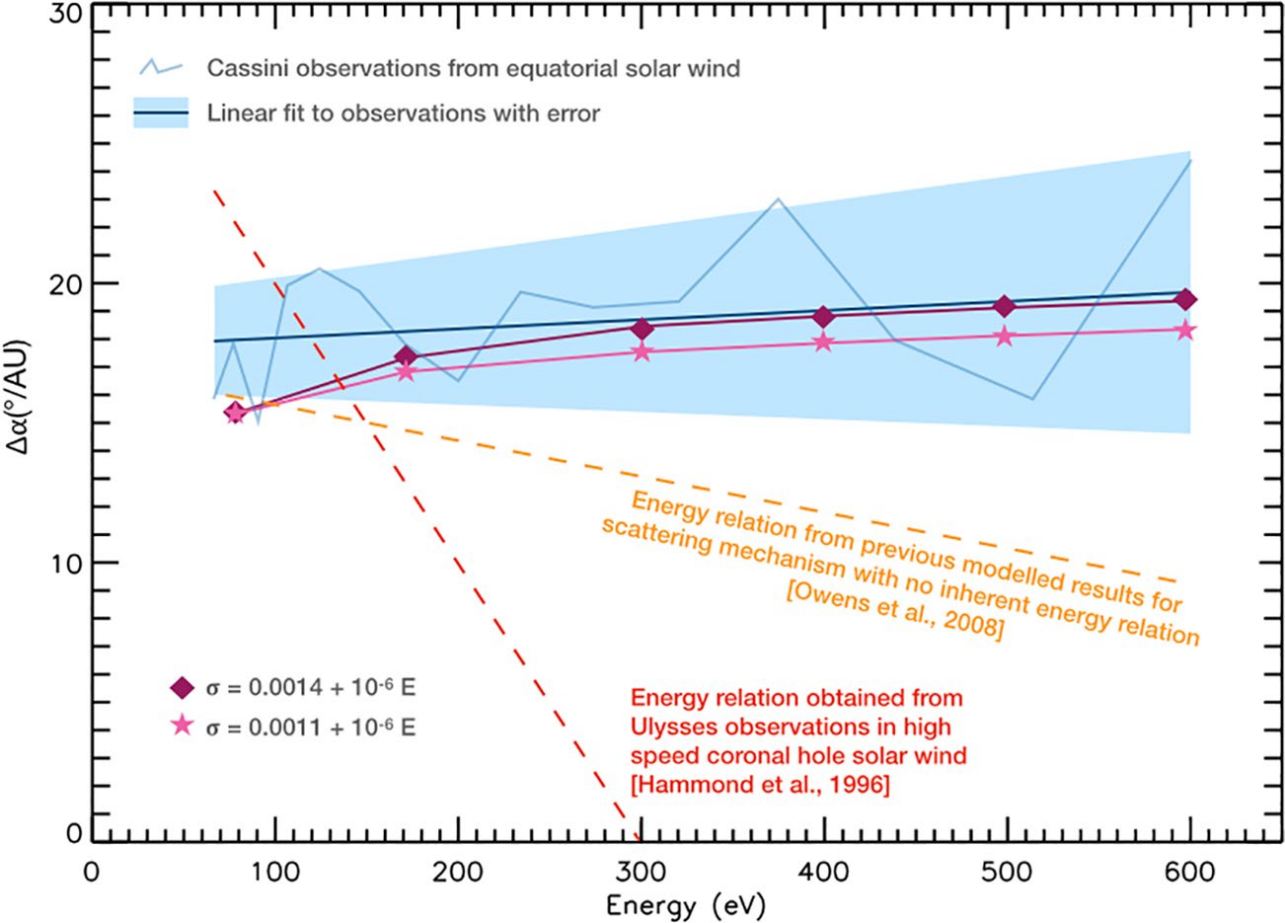
Summary plot showing modeled results from this investigation with observational and modeled results from previous investigations. The increase in strahl width per unit radial distance obtained from Cassini observations is shown by the blue solid line, and the associated uncertainty is shown by the blue shaded area. The increase in strahl width per unit radial distance obtained from Ulysses observations is shown by the red dashed line. The Owens et al. (2008) energy relation for modeled time of flight effects in a Parker spiral field, with a constant scattering factor and a modeled solar wind speed of 800 km^−1^, is shown by the orange dashed line. The purple diamond and pink stars show the simulation results from this investigation. Both are for a scattering factor that increases with electron energy in solar wind with a speed of 450 km^−1^.

In this investigation, we adapted the Owens et al. (2008) model of suprathermal electron evolution, in order to investigate the effect of solar wind speed and a scattering rate that was not constant with electron energy. In particular, the model was adjusted to match the observations made from 1 to 5.5 AU by Graham, Rae, Owen, Walsh, et al. (2017) using Cassini data. Previously, Owens et al. (2008) demonstrated that using a constant scattering factor of 0.0022 produced a good fit between model and the change in strahl width with heliocentric distance observed by Hammond et al. (1996) using Ulysses data. However, Owens et al. (2008) produced an energy relation for pitch angle broadening per AU which did not match the energy relation obtained from the Ulysses observations (see Equation 1). Nor did the modeled results match those obtained by Cassini, which themselves differed significantly from the Ulysses observations. Figure 6 shows the energy relations found by each of these three investigations in addition to two of the modeled results from this study which implemented a scattering factor that increased with electron energy. A primary difference between these two sets of strahl observations is that they were obtained in different solar wind regimes, with Ulysses in the high latitude fast solar wind and Cassini in the low latitude mixed‐speed solar wind. It was concluded that differing solar wind conditions and a scattering mechanism (or mechanisms) with an inherent energy relation may be needed to explain the differences found by the three studies.

We implemented the electron scattering simulation developed by Owens et al. (2008) for a number of simulations with different solar wind velocities, electron energies, and scattering rates. In the initial investigation it was assumed that the scattering rate was constant with time, distance, and electron energy. As expected, it was found that the more tightly wound Parker spiral field, associated with lower solar wind speeds, resulted in a greater strahl width broadening per AU than for a more radial field, associated with faster wind speeds. This is in agreement with findings that strahl is generally broader in the slow solar wind than the fast (e.g., Fitzenreiter et al., 1998). In the case of our modeled results, this greater broadening is a result of electrons traveling further along the spiral field for a given decrease in magnetic field strength and therefore adiabatic focusing effect. In addition, it was found that electrons in the slow solar wind have a steeper electron energy relation for broadening per AU. This steepening is a result of more energetic (faster) strahl electrons experiencing less scattering for a given distance traveled along the IMF, an effect which is more pronounced for more tightly wound, spiral fields.

The Owens et al. (2008) model assumes a Parker spiral field and, although on average the IMF topology agrees with the Parker solar wind model, observations have also shown that the in‐ecliptic magnetic field angle can significantly deviate from the expected spiral field direction (e.g., R. Forsyth et al., 1996). Hence, the variation in strahl beam width observed at a given radial distance (e.g., Anderson et al., 2012; Graham, Rae, Owen, Walsh, et al., 2017, 2018) may in part be explained by the IMF deviation from the spiral field direction. The effect of IMf path length can clearly be observed in our results. In particular, the steepening of the broadening per AU energy relation for simulations with slower solar wind speed (greater IMF length) that can be observed in Panel (d) of Figure 2. This model therefore demonstrates how variation of IMF length can provide significant variation in strahl width at a given radial distance, even without considering the possibility of different scattering mechanisms in the different solar wind regimes.

Previous work, in which the IMF path length traveled by strahl within 1 AU was estimated using SEP onset observations at 1 AU, found that that strahl beam width increased with path length, indicating that strahl scattering is a quasi‐continuous process (Graham, Rae, Owen, & Walsh, 2018). It was also found that the strahl broadening per unit distance estimated within 1 AU was greater than observed at larger distances by Cassini. Path length dependent scattering has also recently been demonstrated in a study of sunward directed strahl observed by the Helios spacecraft (Macneil et al., 2020). The study found that, at a given heliocentric radial distance, sunward strahl was broader than its outward directed counterpart. This result suggests that for a more complex IMF, such as one with local inversions in the field, strahl will travel a longer path along the field to reach a given radial distance and thus experience additional scattering effects. It was also shown that this effect was more pronounced closer to the Sun, suggesting that the relative importance of additional path length dependent scattering decreases with heliocentric distance. For both studies, a constant‐rate scattering process was found to be an appropriate explanation for their observations.

In this investigation, we examined the effect of a scattering factor that remained constant with time and distance but that increased with electron energy. It was found that this form of scattering factor produced an energy relation that agreed well with the best fit to the Cassini observations. It was also found that, when using a scattering factor that increased with electron energy, slower solar wind speeds were a more appropriate match to the Cassini observations. In simulations with faster solar wind speeds, it was found that higher scattering rates were required to match the observed energy relation for strahl broadening per AU. This produced a modeled strahl width at a given radial distance that is broader than observed by Cassini and no longer within error of the Graham, Rae, Owen, Walsh, et al. (2017) radial fits to the observations. Hence, it is concluded that Cassini most likely observed the radial evolution of strahl in predominantly slow solar wind. This is in agreement with the solar wind speeds expected to occur most often in the ecliptic, as well as the solar wind speed estimates made during the Earth and Jupiter flybys (at ∼1 and 5–5.75 AU respectively.)

The energy relation for strahl broadening per unit distance within 1 AU has also been indirectly examined by Graham, Rae, Owen, and Walsh (2018). Indications were found of strahl beam broadening per unit distance that increased with electron energy, in general agreement with the Cassini observations at greater radial distances but with a greater magnitude of beam broadening and a steeper increase in broadening per unit distance. More recently, Helios electron data has been re‐examined to investigate strahl evolution within 1 AU while considering the effect of electron beta (Berčič et al., 2019). It was found that at given radial distance lower beta solar wind, in other words, faster, and more tenuous solar wind, displayed clear energy relations for strahl width; whereas, higher beta winds displayed greater, more uniform strahl widths for all energies. For the lower beta solar wind observed by Helios, lower strahl energies (200 eV) displayed an anti‐correlation with strahl beam width, whereas higher strahl energies displayed a correlation. These two relations are the similar to those obtained using Cassini observations at 1 AU, in which it was found that for lower strahl energies (∼70–150 eV), strahl width decreased with energy, and for higher energies (∼200–600 eV), strahl width increased with energy (Graham, Rae, Owen, Walsh, et al., 2017). The Cassini observations beyond 1 AU generally displayed much less clear or uniform energy relations at a given radial distance. Finally, examination of the Bercic et al. (2019) Helios results indicates that direct observations within 1 AU also show greater strahl beam broadening per unit radial distance for higher electron energies, with magnitudes of beam broadening that generally agree with the indirect observations of Graham, Rae, Owen, and Walsh (2018).

Graham, Rae, Owen, Walsh, et al. (2017) concluded that a possible explanation for the strahl broadening per AU observed by Cassini is that the dominant scattering process is due to resonant interactions with whistler mode waves resulting from turbulent cascade. This conclusion was based on previous simulations of this mechanism, which found that strahl scattering was more effective at higher electron energies (Saito & Gary, 2007b). In this case, strahl broadening with increasing energy is a natural consequence of a turbulent spectrum with greater wave power for longer wavelengths (Saito & Gary, 2007a). However, it should therefore be noted that kinetic Alfvén waves may also be a candidate for strahl scattering, particularly since there have been observations of kinetic Alfvén wave at appropriate scales in the solar wind (e.g., Lacombe, Alexandrova, & Matteini, 2017). Strahl itself could drive instabilities which result in scattering of the strahl beam, particularly for higher strahl energies. A number of possibilities for self‐induced strahl scattering has recently been investigated by Verscharen et al. (2020). This study found that, for low‐beta conditions and sufficiently high strahl speeds, strahl electrons could quasi‐continuously excite the oblique fast‐magnetosonic/whistler instability as the solar wind travels outwards away from the Sun. Thereby, pitch angle scattering the strahl electrons via transfer of kinetic energy into unstable wave modes.

The possible scattering mechanisms highlighted above do not explain the steep decrease in strahl broadening per AU observed by Ulysses in the high speed, polar solar wind (Hammond et al., 1996). Kinetic modeling of strahl electrons which relies on Coulomb collisions as a source of scattering in high speed solar wind streams can produce a strahl width energy relation that falls with electron energy and matches observations at 1 AU (Horaites, Boldyrev, Wilson, et al., 2017). However, the widths of strahl in this type of model saturate at 1 AU and do not become broader with increased heliocentric distance (Horaites, Boldyrev, & Medvedev, 2018). It therefore seems likely that there must be another scattering mechanism(s) acting within the fast solar wind that can then account for continued broadening of the strahl and there are a number of different possibilities. For example, it has been shown that a core electron temperature anisotropy (*T*_*ec*⊥_/*T*_*ec*∥_) > 1 can lead excitation of the whistler anisotropy instability, producing enhanced whistler fluctuations that result in strahl scattering that decreases with strahl energy (Saito & Gary, 2007a). It has also been shown that there are strahl driven processes that can scatter lower energy strahl electrons effectively via either the production of lower hybrid waves (Shevchenko & Galinsky, 2010) or Lagmuir waves (Pavan et al., 2013).

Whistler mode waves are frequently invoked as a scattering mechanism to explain observed strahl beam width broadening, since the waves resonantly interact with suprathermal electrons and they can provide different inherent energy relations depending on their generation mechanism (e.g., Anderson et al., 2012; De Koning et al., 2006; Fitzenreiter et al., 1998; Hammond et al., 1996; Pagel et al., 2007; Vocks et al., 2005). It is therefore important to consider the surrounding conditions and properties of the whistler waves that are observed in the solar wind. Whistler waves have been observed in the solar wind at 1 AU by a number of different investigations. For example, it has been shown that whistler‐like fluctuations are present in the solar wind up to 10% of the time, in particular when the wind has a slow speed (<450 km/s), a relatively large electron heat flux, and a low electron collision frequency (e.g., Lacombe, Alexandrova, Matteini, et al., 2014). Although, it has also been shown that the majority of whistler mode waves observed at 1 AU propagate in the anti‐sunward direction and a sunward propagation direction is required for resonant interaction with anti‐sunward strahl (Stansby et al., 2016).

More recently, it has been shown that the occurrence probability of whistler waves in the solar wind is strongly dependent on the electron temperature anisotropy (Tong et al., 2019). When *T*_*e*⊥_/*T*_*e*∥_ < 0.9 the probability is less than 2% but this increases to 15% as *T*_*e*⊥_/*T*_*e*∥_ approaches 1.2. This particular investigation of whistler waves also found that the wave amplitude anti‐correlates with solar wind velocity and strongly correlates with electron beta. Additionally, the minimum energy of electrons resonating with the whistler waves was found to increase with decreasing electron beta, from a few tens of eV to a few hundred eV. Finally, whistler wave packets have also recently been observed in the solar wind within 1 AU by the Parker Solar Probe spacecraft (Agapitov et al., 2020). It was found that the waves propagated in the sunward direction necessary to interact with strahl beams and that the waves had much larger amplitudes than observed at 1 AU.

## 6 Conclusions

The simulated results obtained in this study show that the large scale IMF path associated with slow solar wind speeds provide the best match to the strahl widths observed by Cassini. This agrees well with the expected conditions observed by Cassini in the elliptic plane of mixed, mostly slow, solar wind velocities. It is also possible that differing solar wind conditions may explain the opposite strahl broadening energy relations obtained using the Cassini and Ulysses observations (see Equations 2 and 3, respectively). The Ulysses observations were made in coronal hole solar wind and thus not only have shorter average IMF path lengths at a given radial distance, as a result of high solar wind speeds; but also different plasma properties, which may result in a different dominant scattering mechanism. These different plasma conditions are beyond the scope of this study but many recent studies have explored the effect of differing electron beta and electron velocity distribution anisotropies. In particular, the Parker Solar Probe and Solar Orbiter spacecraft will enable these kinds of investigations in regions close to the Sun, where much less in‐transit processing has occurred and the coronal influence on the observed velocity distributions may be established (e.g., Berčič et al., 2020; Halekas et al., 2020)

In this investigation, it was found that linear fitting to the modeled increase in strahl width with distance for each electron energy, in order to determine the energy relation for strahl broadening per AU, is appropriate for higher energy strahl electrons. However, the modeled broadening of strahl electrons follows only an approximately linear trend and thus, when considering a large radial range, this is not suitable for use with lower energy strahl. Higher energy electrons do not experience as significant a decrease in strahl broadening per AU as their lower energy counterparts and, for these energies, it was found that a scattering factor that increased with strahl energy produced an energy relation for strahl broadening per AU that closely matched the Graham, Rae, Owen, Walsh, et al. (2017) observations. The results presented in this investigation suggest that the geometric effect of different solar wind speeds, that is, the IMF length variation at a given radial distance, can account for some of the strahl width variation observed. However, it is found that the strahl broadening energy relation cannot be explained by differing solar wind speeds and that an inherent non constant scattering rate which increases with energy is required to match the Graham, Rae, Owen, Walsh, et al. (2017) results. Thus, it is concluded that the dominant strahl scattering mechanism in the ecliptic solar wind must have an inherent energy relation.

Finally, it should be noted that the scattering factor used in this investigation is “ad‐hoc.” Furthermore, high resolution, investigation of individual strahl scattering events at a given radial distance are needed to ascertain the degree by which strahl is pitch angle broadened and to determine the scattering event occurrence. This would not only provide constraints by which the dominant strahl mechanism at that radial distance could be identified but also mean that a scattering factor based on observational evidence could be implemented in the Owens et al. (2008) model for strahl evolution.

## Data Availability

Data for this study can be found at NASA's Planetary Data System (https://pds.jpl.nasa.gov).
